# A Short-Term Pacing Intervention in People with Myalgic Encephalomyelitis/Chronic Fatigue Syndrome: A Pilot Study in Portugal

**DOI:** 10.3390/medicina62020331

**Published:** 2026-02-06

**Authors:** Vânia Ribeiro, Paulo Azevedo, Francisco Westermeier, Nuno Sepúlveda

**Affiliations:** 1 Escola Superior de Saúde Norte da Cruz Vermelha Portuguesa, 3720-126 Oliveira de Azeméis, Portugal; paulo.azevedo@essnortecvp.pt; 2 Department of Health Studies, Institute of Biomedical Science, FH Joanneum University of Applied Sciences, 8020 Graz, Austria; francisco.westermeier@fh-joanneum.at; 3 Centro de Biología y Química Aplicada (CIBQA), Universidad Bernardo O′Higgins, Santiago 8370993, Chile; 4 Faculty of Mathematics & Information Science, Warsaw University of Technology, 00-662 Warsaw, Poland; 5 CEAUL—Centro de Estatística e Aplicações, Faculdade de Ciências, Universidade de Lisboa, 1749-016 Lisboa, Portugal

**Keywords:** ME/CFS, pacing, energy management, fatigue, physical functioning, quality of life

## Abstract

*Background and Objectives:* Myalgic Encephalomyelitis/Chronic Fatigue Syndrome (ME/CFS) remains a disease without a curative treatment. Hence, patient healthcare is mostly based on symptom management and the application of coping strategies, such as pacing. In this strategy, patients learn how to plan their daily physical and cognitive activities according to their perceived energy reservoir (or envelop). However, there is currently no evidence for the feasibility of pacing in Portugal, where ME/CFS is not well recognized. *Materials and Methods:* We implemented a 8-week pacing program in Portuguese patients with an official diagnosis of ME/CFS. We focused on recruitment feasibility, protocol adherence, and patient acceptability, with secondary exploratory analysis of pre- and post-intervention variations in the Chalder’s fatigue questionnaire and SF36 physical functioning scores. *Results:* We were able to recruit thirteen patients for the study. The patients attended, on average, seven out of the eight sessions expected per participant, with the majority adhering to the research protocol (n=7;53.8%). In a post-intervention survey, the respondents (n=10) considered that the intervention addressed the specific needs of people living with ME/CFS. Concerning the outcome trends, the average fatigue score decreased from 27.5 at baseline to 17.7 after the intervention. The mean physical functioning score increased from 24.6 to 31.7. *Conclusions:* This exploratory study supported the feasibility of benchmark studies in Portugal with increased sample size, longer interventions, and including a control group (e.g., specialized medical care), with which eventual placebo effects can be better accounted for.

## 1. Introduction

Myalgic Encephalomyelitis/Chronic Fatigue Syndrome (ME/CFS) is a complex and multifactorial disease without an objective biomarker for the respective diagnoses. As such, clinicians rely on the application of consensus case definitions to suspected cases. These case definitions, such as the Canadian Consensus Criteria or the modified Center for Disease Control criteria, are based on unexplained fatigue for more than 6 months and post-exertional malaise (PEM) as cardinal symptoms of the disease [1,2,3]. The pathogenesis of the disease remains unclear; however, in patients who reported a microbial infection (e.g., Epstein–Barr virus, Ross river virus or Borrelia bacteria) at symptom onset, there is evidence of pathological autoimmunity driven by auto-antibodies against adrenergic receptor antigens [4,5]. This evidence prompted the research community to test treatments targeting the removal of these auto-antibodies [6,7,8,9,10,11]. However, these interventions seemed no better than a placebo [8] or remain to be tested in larger cohorts of patients [10,11].

Given the absence of a curative pharmacological treatment and the frequent unavailability of ME/CFS experts in primary care practices [12,13], patients rely on self-care. In this regard, a recent study conducted a large-scale survey about the different disease management strategies adopted by patients and their impact on perceived health outcomes [14]. One of the most popular strategies is so-called pacing/energy management therapy, as reviewed elsewhere [2,15].

Pacing is based on the energy envelop theory in which patients are thought to have a fixed amount of energy per day (i.e., the energy envelop) [16]. As such, patients should learn how to plan their daily physical and cognitive activities according to their perceived energy envelop. By maintaining the energy levels within the envelop, patients are expected to avoid the onset of PEM [3]. However, a recent systematic review and meta-analysis provided limited evidence for the potential benefit of pacing decreasing fatigue and pain levels and increasing physical functioning when compared to a control group, such as patients receiving specialized medical care or utilizing relaxation techniques [17].

Notwithstanding the potential clinical benefits of pacing, it is unclear whether such a non-pharmacological intervention is widely accepted by patients [18], especially in countries in which the disease has little public and professional recognition. This is the case of countries like Ireland [19] and Portugal [20], where there are no recommended disease management strategies at the level of public health policy. In the case of Ireland, clinicians tend to follow the United Kingdom’s NICE guidelines [19], where pacing is seen as one of the general management strategies.

With respect to Portugal, there are no studies addressing the feasibility and acceptability of a pacing-based intervention in people living with ME/CFS. To fill in this research gap, we conducted a pilot study utilizing a short-term pacing intervention in a cohort of 13 Portuguese patients with ME/CFS.

## 2. Materials and Methods

### 2.1. Study Design and Context

The study was conducted as a Master’s thesis in Rehabilitation Nursing at the *Escola Superior de Saúde Norte da Cruz Vermelha Portuguesa*, Portugal. The initial study design (e.g., exploratory/pilot nature, lack of funding, minimal patient risk, Institutional Review Board not initially requiring registration) did not justify the prospective registration of the research protocol. However, once publication became viable, we conducted a retrospective registration of the study in the ClinicalTrials.gov platform (ref. NCT07363655; date of registration: 23 January 2026). All methods and outcomes are fully described here.

Focusing on feasibility and acceptability specifically, the study was designed as a single-group, two-sample evaluation, with the first at baseline and the second at the end of the intervention. This group received a short-term pacing intervention (8 weekly sessions of pacing).

Given this was the first study on people with ME/CFS in Portugal, we designed the study to address recruitment feasibility, intervention delivery, protocol adherence, and patient acceptability, with the analysis of outcome trends as exploratory. As such, we expected to recruit no more than 20 patients, without any formal sample size determination and power calculation, supporting the design of the study. In other words, we did not pre-specify any minimal clinically important difference between pre- and post-intervention.

Therefore, this study should be seen as exploratory or descriptive, as in single-arm clinical trials [21].

### 2.2. Recruitment and Enrollment

Recruitment was performed on 9–17 October 2024, using social media platforms and contacts with two Portuguese patients’ associations: *EM-Movimento* and *MYOS–Associação Nacional Contra a Fibromialgia e Síndrome de Fadiga Crónica*. The individuals willing to participate were then contacted by phone and informed of the conditions to participate in the study, including a short description of the intervention and the respective means of delivery.

The inclusion criteria were the following: (i) Portuguese-speaking individuals 18 years of age or older; (ii) individuals with official residence in Portugal; (iii) an official diagnosis of ME/CFS based on the NICE guidelines, as reviewed in Kingdon et al. [22] (see also https://www.nice.org.uk/guidance/ng206/chapter/recommendations#diagnosis (accessed on 9 August 2024)); and (iv) a Likert-based Chalder’s Fatigue Questionnaire (CFQ) score higher than 18 points, as specified in the PACE trial for patients’ recruitment [23].

Note that the existence of an official diagnosis of ME/CFS was self-reported, given that we neither accessed the respective health records nor performed any clinical or lab testing. To verify the NICE-based diagnosis as much as possible, we asked the participants about the specialty of the physician who diagnosed them and which diagnostic criteria were used by the physician. We also asked whether participants suffered from all four core conditions of NICE-based criteria: (i) debilitating fatigue that is worsened by activity, is not caused by excessive cognitive, physical, emotional or social exertion, and is not significantly relieved by rest; (ii) PEM; (iii) unrefreshing sleep; and (iv) cognitive difficulties (sometimes described as ’brain fog’), which may include problems finding words or numbers, difficulty in speaking, slowed responsiveness, short-term memory problems, and difficulty concentrating or multitasking. Finally, we asked whether the participants suffered from any clinical condition which could explain their symptoms. The respective questionnaires (in Portuguese) can be found in the Supplementary File.

Note also that a value of 18 for the Likert-based CFQ score is optimal in distinguishing people with ME/CFS from the United Kingdom’s general population [24]. This cut-off point is associated with a value of 85% for both sensitivity and specificity.

The exclusion criteria were the following: (i) individuals without official residence in Portugal; (ii) patients without an official diagnosis of ME/CFS; (iii) patients without a diagnosis of ME/CFS who had previously participated in any kind of rehabilitation programme, including pacing, cognitive behavioural or graded-exercise therapies.

**Figure 1:**
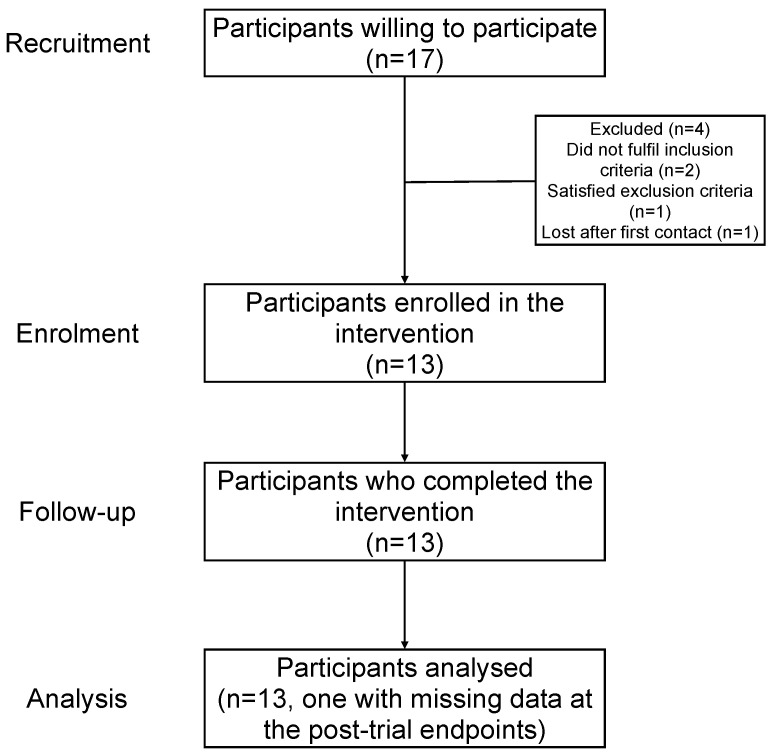
Flowchart of the study.

Figure 1 shows the flowchart of the study from recruitment to analysis.

### 2.3. Pacing Intervention: Content and Delivery

Given the absence of official guidelines for diagnosis and treatment of ME/CFS in Portugal [20], the intervention consisted of an educational program focused on energy management and developed using different sources (Supplementary Table S1), including the United Kingdom’s NICE disease management guidelines (https://www.nice.org.uk/guidance/ng206 (accessed on 9 August 2024)). The program encompassed a single group session per week for eight consecutive weeks. Each session was delivered online (Microsoft Teams) at the same time (16h00) with an average duration of 60 min.

The content of the program was based on the progressive acquisition of knowledge about ME/CFS and pacing as a self-care strategy (see detailed information in Supplementary File (Supplementary Table S1)). The overall aim of the intervention was the empowerment of patients with self-management strategies that could help them to maximize their autonomy, independence and daily activities without exceeding their perceived energy reservoir. The program highlighted the importance of having a sustainable balance between periods of activity (physical or cognitive) and of rest.

The program started with sessions on general knowledge of the disease, including an overview of its putative pathophysiological mechanisms, its symptomatology, the functional impact and putative therapies. After that, the topic related to PEM was addressed.

In following sessions, the participants learned the basic principles and techniques associated with energy management, receiving a template diary for their daily activities and symptoms. They were also exposed to a series of slide shows with additional tools for energy conservation, as well as a digital tool for monitoring their heart rate to be used as means of identifying possible episodes of flare-ups and exhaustion.

Participants were then given information about the factors that commonly influence the energy levels, such as sleep, nutrition, hydration, and emotional management.

The focus of one of the sessions was on communication skills (e.g., assertiveness) that allow patients to communicate their needs better to caregivers and health professionals. Participants were encouraged to develop these communication skills as a way of building (or strengthening) trust and emotional support among all the parties involved in the disease management process.

The last session focused on the long-term planning that would allow the participants to envision their gradual return to work or school, using all the knowledge provided.

All the sessions were delivered by the first author (VR) who is a nurse specialist in rehabilitation nursing. The sessions were conducted from 18 October to 6 December 2024. No data was collected concerning adverse events or adverse reactions to the intervention.

### 2.4. Assessing Protocol Adherence and Patient Acceptability

To assess protocol adherence, we recorded the attendance of each session and compiled the number of sessions attended by each participant. We also calculated the number of participants who attended the expected number of sessions during the intervention.

To address patient acceptability, patients were first asked an open question about their expectations before the delivery of the intervention. The respective answers were analyzed and grouped into four main themes: (i) improvement in daily functioning and quality of life; (ii) improvement in managing the disease/symptoms/energy; (iii) increase in the information and education about the disease; and (iv) no expectations or unsure about what to expect.

A survey of the participants was conducted at the end of the study to assess the overall experience of the participants (categories of responses: bad, not good/not bad, good, very good, excellent) and to understand whether the main challenges of people living with ME/CFS were addressed in the intervention (categories of responses: not addressed, poorly addressed, partially addressed, and completely addressed).

### 2.5. Exploratory Interventional Outcomes

We performed an exploratory analysis focusing on pre- and post-intervention variation in the Likert-based CFQ score, the SF36-Physical Functioning domain (SF36-PF) and quality of life. In theory, the CFQ score varies from 0 (no perception of fatigue) to 33 points (maximum perceived fatigue). We calculated this score via a Brazilian version of the questionnaire [25], adapted to Portuguese from Portugal. With respect to SF36-PF score, we used the SF36-v2 questionnaire that was previously validated in the Portuguese population [26,27]. We evaluated the quality of life using the Portuguese version of EQ-5D-5L [28]; the use of this questionnaire was approved by the EuroQol Research Foundation (ID 65399).

All the questionnaires were filled in by the participants using online forms or by the interviewer (VR) if requested.

All of these outcomes were evaluated at baseline and twelve weeks after the last pacing session.

### 2.6. Statistical Analysis

We performed exploratory and descriptive statistical analysis, given that this study followed a uncontrolled, single-arm design, as recommended for this type of study [21]. Similar to a single-arm clinical trial, we have used statistical analyses that are exploratory and descriptive. It should be noted that the data comes from a conveniently recruited cohort of patients, thus violating the assumption of a random sample underlying statistical inference [29]. As such, we calculated means, medians, and standard deviations (SD) for summarizing quantitative data. We also calculated standardized mean difference (SMD) between pre- and post-trial outcomes (i.e., mean difference between post- and pre-intervention means divided by the respective standard deviation) [30]. The use of this dimensionless metric allowed us to compare the variation in the different endpoints before and after the intervention.

We used the Spearman’s correlation coefficient (*R*) to assess the statistical relationship between pre- and post-intervention outcomes. Finally, for qualitative socio-economic, demographic variables, we used frequencies, proportions, and percentages.

We had post-intervention missing data regarding CFQ, SF36-PF, and EQ-5D-5L scores from a single individual (Figure 1). Given that these three outcomes were correlated with each other, we took advantage of this statistical fact and performed multiple imputation based on chained equations (MICE), as available in the package mice for the R software [31].

Our MICE procedure was based on predictive mean matching using a joint imputation model including pre- and post-intervention data regarding CFQ, SF36-PF, and EQ-5D-5L scores together and the number of sessions attended per participant. The length of the chains associated with the algorithm was fixed at 5 (default option), while the number of imputed datasets was 10. To pool estimates from imputed datasets, we calculated the respective mean of the statistical indicator under analysis.

All the analyses were made in the R software (Vienna, Austria), version 4.5.1. The respective scripts are available from the corresponding authors upon requested.

## 3. Results

### 3.1. Basic Characterization of Study Participants

Thirteen participants were enrolled to receive the intervention (Figure 1). Twelve were female and only one was male (Table 1). The age ranged from 18 to 60 years old, with an average of 40.1 and an SD of 13.5 years. The majority of participants had an age between 18 and 39 years old.

There was almost an equal proportion of participants with a high school or a university degree. Four participants were married. The same frequency was observed for the civil statuses of “single” and ”other”.

In terms of professional status, the majority of participants (n=7; 53.8%) were on sick leave. Three participants were unemployed. Three participants conducted some form of work, such as a student (n=2;15.4%) or as a part-time worker (n=1; 7.7%).

**Figure 2:**
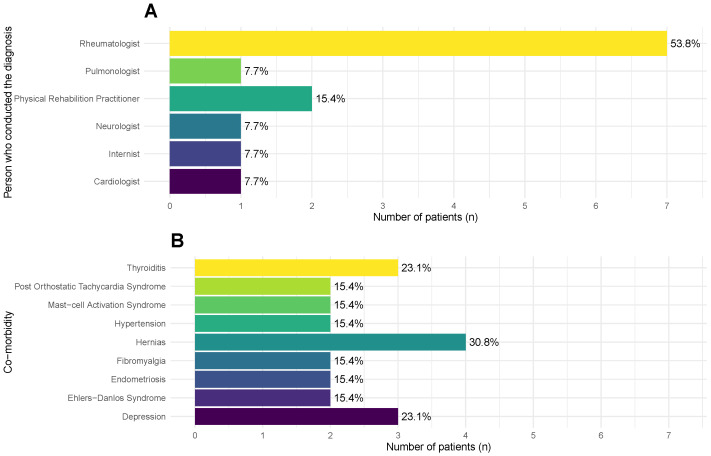
Frequencies and percentages of different medical specialists who were responsible for the diagnosis of ME/CFS (**A**) and of the main co-morbidities (**B**) reported by the participants (*n* = 13) at the beginning of the study (see main text for the complete list of co-morbidities).

According to the participants, their ME/CFS diagnosis was mostly conducted by rheumatologists (n=7, 53.8%) (Figure 2A). Other physicians conferring diagnoses were physicians specialized in physical and rehabilitation medicine (n=2, 15.4%), a neurologist (n=1, 7.7%), a pulmonologist (n=1, 7.7%), an internist (n=1, 7.7%), and a cardiologist (n=1, 7.7%). Eight diagnoses were conducted in private clinics, while the remaining five were made at the Portuguese National Health Service (*Serviço Nacional de Saúde*).

Overall, the participants self-reported many co-morbidities. In Figure 2B, we reported the co-morbidities with a frequency above 1; note that each participant reported more than one co-morbidity. The most frequent one was hernias (n=4, 30.8%), followed by depression and thyroiditis (n=3; 23.1%). Other co-morbidities were reported by individual participants: osteopenia, erosive nodal osteoarthritis, carpal tunnel syndrome, food allergies, asthma, chronic gastritis, irritable bowel disease, small-fibre neuropathy, restless-leg syndrome, tachycardia, myopia, and polycystic ovary syndrome.

Before the beginning of the intervention, every participant was asked about their prior expectations (Table 2). Most of the participants had multiple expectations, but three of them mentioned that they had no expectations or were unsure of what to anticipate. The most frequent expectation was a better management of the disease/symptoms/energy (n=5), which was in alignment with the global objective of the intervention. Interestingly, two participants intended to increase disease awareness/education.

### 3.2. Analysis of Protocol Adherence and Intervention Acceptability

The average number of sessions attended per participant was seven out of the expected eight weekly sessions. More importantly, the majority of patients (n=7, 53.8%) followed the protocol. The most extreme case was one participant who only attended two sessions during the study period.

There was one participant who did not provide their assessment of the post-trial endpoint questionnaire. Missing data from this individual was filled in with multiple imputation based on chained equations.

At the end of the trial, we conducted a short survey about the degree of satisfaction with the intervention. Ten participants responded to the survey, while three did not. Nine respondents answered that the overall experience of the intervention was excellent. The remaining participant considered the overall intervention very good. The ten respondents were unanimous in declaring that the content of the intervention addressed completely the main challenges of people living with ME/CFS.

Given the little variation in the responses received, we decided to not perform any imputation on the missing data of the three patients who did not respond to the survey.

### 3.3. Exploratory Analysis of Outcome Trends

**Figure 3:**
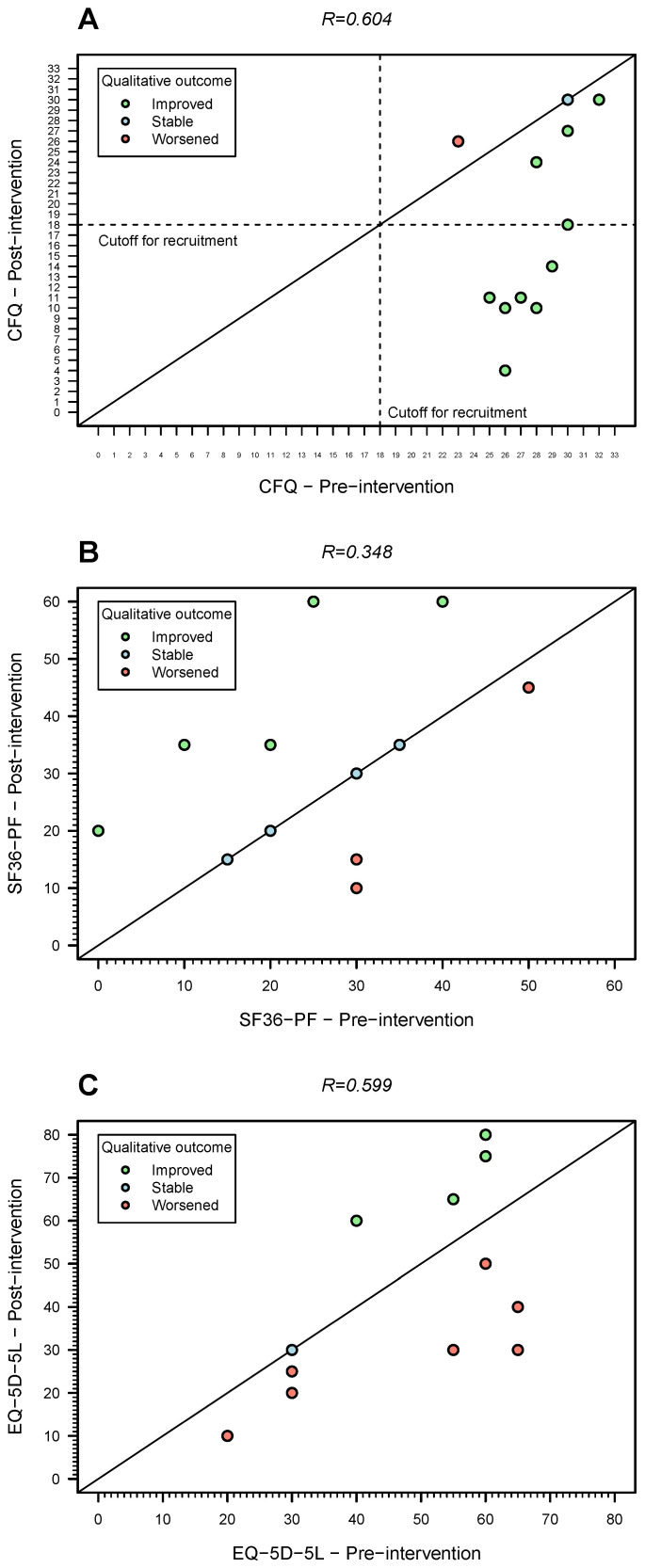
Pre- and post-intervention outcome trends for the 12 patients who responded to the final evaluation questionnaire. (**A**) Fatigue (Likert-based CFQ score; the dashed lines represent the cutoff used for recruitment); (**B**) physical functioning (SF36-PF score); (**C**) quality of life (EQ-5D-5L, secondary endpoint). The diagonal solid lines in three plots represent the situation in which the value of a given metric did not change between pre- and post-intervention. Note that this figure does not include one patient who had missing data on the post-trial outcome. The Spearman’s correlation coefficient (*R*) estimates shown are for complete cases only.

The average of the Likert-based CFQ score decreased from 27.5 (SD = 2.7) to 17.7 (SD = 8.9), an average reduction of almost 10 points after the intervention. Around 50% of the patients reported a Likert-based CFQ score lower than 18 points, the cut-off used at the recruitment stage. There were only two patients (15.4%) who reported stable or worsening of their perception of fatigue after the intervention (Figure 3A). For the individual who had a missing post-intervention Likert-based CFQ score, the imputation model predicted an average improvement of 9.4 points, in agreement with the predictive mean matching method for data imputation.

Note that the same imputation model predicted 2 instances out of 10 data imputations in which this individual had a worsening of fatigue levels from baseline to post-intervention (from 24 at baseline to 26 or 27 points after intervention).

The average SF36-PF score increased from 24.6 (SD = 13.3) to 31.7 points (SD = 16.7) after the intervention (Table 3). In more detail, there were five patients (38.5%) who reported an improvement in their physical functioning after the intervention (Figure 3B). Four patients (30.8%) reported no change in this endpoint, while three patients (23.1%) perceived a worsening of their physical functioning (Figure 3B).

For the individual who had a missing post-trial SF-36-PF score, the imputation model predicted an average increase of 17.5 points in this score. The same model predicted 1 instance out of 10 data imputations performed in which this individual had a worsening in their physical functioning (from 15 at baseline to 10 points after intervention).

The mean quality-of-life (EQ-5D-5L) score decreased from 45.8 (SD = 16.8) to 42.1 (SD = 22.2), an average decrease of 3.7 points. This score increased, remained stable or decreased in four, one, and seven individuals, respectively (Figure 3C). For the individual who had a missing post-trial EQ-5D-5L, the imputation model predicted an average increase of 7 points. Three instances out of ten data imputation performed predicted a post-trial value less than the pre-trial one (from 25 to 20).

To understand the findings better, we calculated the correlation matrix between the primary and secondary endpoints before and after the intervention (Table 4). At the baseline, all the endpoints were slightly correlated (|R|<0.33). This contrasted with the moderate to strong correlations in absolute terms among the same endpoints after the intervention. The finding suggests that the statistical relationship among the three endpoints changed at the end of intervention.

## 4. Discussion

The study suggested that pacing was feasible and well tolerated by the 13 participants enrolled in the study. Ten participants who responded to the survey found the intervention helpful.

Exploratory analysis of the outcomes showed a trend toward reduced fatigue scores and increased physical functioning after the intervention. However, this variation should be interpreted with caution, because it might be explained by not only the genuine effect of the intervention, but also by placebo effects, natural variation in disease, a regression to the mean, or heterogeneity in disease triggers, including infections caused by different microbes. This caution is due to the uncontrolled, single-arm study design that cannot address efficacy without including a control group.

To ascertain a genuine intervention effect, a follow-up study should consider the inclusion of a control group in the study design. In this regard, control groups could be specialized medical care or a relaxation group, as reviewed elsewhere [32]. Longer follow-up should be considered; however, it would increase the costs of a given study. Longer follow-up in a pacing-related study was two years after treatment allocation in the controversial PACE trial [33,34]. In Portugal, where the research funding is limited, a feasible follow-up time would be around six months. A study with a larger cohort would provide stronger statistical support for the efficacy of pacing on patient-related outcomes. However, the scarce disease recognition in Portugal precludes the execution of studies with larger cohorts. To address this point, it would be important to make efforts to create a list of patients that could be enrolled in this type of study. An alternative is that the Portuguese patients join future international research initiatives in the investigation of the eventual benefits of pacing in disease management. As far as we know, we are not aware of any international initiative of that kind.

Another limitation of this study is that the diagnosis of ME/CFS was self-reported. We tried to minimize this limitation by only enrolling patients with an ”official” diagnosis made by a healthcare professional. We also minimized this limitation by asking participants whether they suffered from all the symptoms complying with NICE case definition. Ideally, the rigorous assessment of the diagnosis requires a critical review of clinical history and extensive clinical and lab testing, as performed in the recruitment of patients for the United Kingdom’s ME/CFS biobank. However, given the exploratory nature of the present study and the lack of available funding, a comprehensive diagnostic assessment was deemed unfeasible by us. According to the participants, the diagnoses of ME/CFS were made by different medical experts, especially rheumatologists. This is far from surprising given that ME/CFS is included in Portugal under the national plan against rheumatic diseases [20]. In Switzerland, for example, a survey showed that it is difficult to obtain an official diagnosis with 13.5% of the 169 surveyed patients traveling abroad for that purpose [35]. For those diagnosed in Switzerland, diagnosis was mostly carried out in clinics specialized in general medicine (34%) and by psychiatrists (32.6%). Interestingly, given that ME/CFS is considered by the World Health Organization as a neurological condition, only one patient from our cohort was diagnosed by a neurologist. In Switzerland, the situation is not much better, with only one in four patients reporting a diagnosis by this medical expert [35].

Despite the small number of participants in our study, data suggests that our cohort is typical of larger ME/CFS cohorts. For example, the average Likert-based CFQ of our cohort at baseline (≈28 points) sits slightly above the 70% percentile of the Likert-based CFQ score distribution for the United Kingdom’s ME/CFS patients (see Table 3 of Cella et al. [24]). Furthermore, the minimum and maximum CFQ values of our study at baseline (23 and 32, respectively) are located in the 40% and 94% percentiles in the distribution for the same population.

In the case of SF36-PF, the average of our cohort is almost in perfect alignment with the one for the participants in the United Kingdom’s ME/CFS biobank [36]. In addition, the maximum value observed in our cohort was 50 points in the SF36-PF at baseline. As such, our cohort would be considered eligible, for example, to participate in the PACE trial in which the efficacy and acceptability of pacing was also tested [23]. According to the protocol used in this trial, the SF36-PF cut-off for recruitment was 65 points or less [37].

In our exploratory analysis, we focused on typical outcomes for evaluating the effect of pacing on people living with ME/CFS. The choice of these outcomes was pragmatic due to the pilot nature of the study. However, a more pacing-specific outcome could have been chosen. In fact, recent ME/CFS studies focusing on this type of intervention used the so-called activity pacing questionnaire [38] as an endpoint, in addition to the classical ones on perceived fatigue levels, physical functioning, and quality of life [32]. The current version of this questionnaire consists of a set of 28 questions concerning five domains: activity adjustment, activity planning, activity consistency, activity progression, and activity acceptance [39]. This questionnaire was already validated in an English-speaking population, but not validated in Portugal. Therefore, the use of this tool requires a prior testing/validation study before its application to the Portuguese patient population.

## 5. Conclusions

In summary, this is the first study conducted in Portuguese people living with ME/CFS. Some limitations were highlighted in terms of study design (without a control group), disease diagnosis (without confirmation) and patient selection (recruitment via patients’ associations), because the study was more focused on feasibility and acceptability than establishing treatment efficacy. For that specific purpose, one should perform a randomized clinical trial using a control group, as reviewed elsewhere [17]. Longer follow-up times should also be considered. Data suggested by these studies merits consideration when drafting science-based healthcare guidelines for Portuguese patients who have been neglected over the years by the national public health authorities. Finally, we hope this study stimulates further interest and research on ME/CFS in Portugal.

## Figures and Tables

**Table 1:** Basic characteristics of study participants (*n* = 13).

Variable	*n* (%)	Variable	*n* (%)
Sex		Civil state	
Female	12 (92.3)	Married	4 (30.8)
Male	1 (7.7%)	Divorced	1 (7.7)
Age group (in years)		Single	4 (30.8)
18–39	7 (53.8)	Other	4 (30.8)
40–59	5 (38.5)	Professional Status	
≥60	1 (7.7)	Sick leave	7 (53.8)
Academic qualifications		Unemployed	3 (23.1)
High school	7 (53.8)	Part-time worker	1 (7.7)
University degree	6 (46.2)	Student	2 (15.4)

**Table 2:** Themes and the respective frequencies concerning the prior expectations of participants enrolled in the study (n=13), noting that some participants had more than one expectation.

Themes of Expectations	*n* (%)
Better management of the disease/symptoms/energy	5 (38.5%)
No expectations or unsure on what to expect	3 (23.1%)
Improvement in daily functioning/autonomy/quality of life	2 (15.4%)
Increase disease awareness/education	2 (15.4%)

**Table 3:** Mean (and standard deviations) of outcomes at baseline and 12 weeks after the end of the intervention (n=13). Note that the post-trial estimates were calculated via data imputation.

Outcome	Baseline	End	End-Baseline	SMD
Likert-based CFQ	27.5 (2.7)	17.7 (8.9)	−9.9 (8)	−1.231
SF36-PF	24.6 (13.3)	31.7 (16.7)	7.1 (16.8)	0.422
EQ-5D-5L	45.8 (16.8)	42.1 (22.2)	−3.7 (18.1)	−0.204

**Table 4:** Spearman’s correlation matrix between outcomes, where the values in the upper and lower diagonal refer to the correlations calculated for pre- and post-intervention data, respectively. Note that post-intervention correlations were estimated via data imputation.

Outcomes	Likert-Based CFQ	SF36-PF	EQ-5D-5L
Likert-based CFQ	1.000	−0.117	0.333
SF36-PF	−0.470	1.000	0.162
EQ-5D-5L	−0.764	0.373	1.000

## Data Availability

The original contributions presented in this study are included in the article/Supplementary Material. Further inquiries can be directed to the corresponding authors.

## Supplementary Materials

The following supporting information can be downloaded at https://www.mdpi.com/article/10.3390/medicina62020331/s1.

## Abbreviations

The following abbreviations are used in this manuscript:

CFQ	Chalder Fatigue Questionnaire
ME/CFS	Myalgic Encephalomyelitis/Chronic Fatigue Syndrome
NICE	National Institute for Clinical Excellence (United Kingdom’s)
PEM	Post-exertional malaise
SF36-PF	Physical functioning domain of the short-form 36 questionnaire
SMD	Standardized mean difference
