# Supplementary material for: A Short-Term Pacing Intervention in People with Myalgic Encephalomyelitis/Chronic Fatigue Syndrome: A Pilot Study in Portugal
Source: Medicina (Kaunas). 2026 Feb 6;62(2):331. doi: 10.3390/medicina62020331 (PMC12941993; doi:10.3390/medicina62020331)
Supplement: Supplementary file 1 [file medicina-62-00331-s001.zip › medicina-4074475-supplementary/medicina-4074475-supplementary Table S1.pdf]

Supplementary Table S1

Detailed information about the theme/objective/main contents for each of the 8 pacing sessions consisting of the intervention (see references after the table).

Session	Theme	Objectives	Main contents
1	Understanding Myalgic Encephalomyelitis/Chronic Fatigue Syndrome (ME/SFC): What is it and how does it affect us?	• Understand ME/CFS, including its main symptoms, diagnostic criteria and levels of severity.<br>• Identify the role of rehabilitation nursing within an interdisciplinary, non-pharmacological approach to ME/CFS management.<br>• Recognise energy management as a key rehabilitation strategy in the overall treatment framework of ME/CFS.	• Historical overview (evolution of understanding of the disease) and context<br>• Etiology<br>• Pathophysiology<br>• Diagnosis (different diagnostic criteria) and symptoms<br>• ME/CFS: Levels of severity<br>• Treatment<br>– General treatment framework in ME/CFS<br>– Introduction to rehabilitation and self-management approaches
2	Post-exertional malaise (PEM): What is it and how can it be managed?	• Understand post-exertional malaise (PEM) as a key feature of ME/CFS.<br>• Identify triggers, manifestations and warning signs of PEM.<br>• Recognise general response principles to PEM episodes.<br>• Enhance awareness of individual PEM patterns through home-based symptom self-monitoring.	• What is Post-Exertional Malaise (PEM)?<br>• PEM – Why does it happen?<br>• PEM – clinical and functional assessment of PEM.<br>• PEM – Warning signs!<br>• PEM – What to do?<br>• "To manage it better, I need to know myself!": Home-based self-monitoring task in which participants were asked to record and reflect on PEM-related symptoms during the week following the session.
3	Our Energy: How Does the Body Acquire and Use It?	• Understand basic principles of energy acquisition, production and expenditure in the human body.<br>• Recognise how daily activities (physical and mental) influence energy use.<br>• Understand metabolic alterations described in ME/CFS at a conceptual level.<br>• Introduce the concepts of energy management and individual energy baseline.	• The Importance of Energy for the Human Body<br>• From Food to Useful Energy: The Role of Mitochondria?<br>• Where Does Our Energy Come From?<br>• How Does the Body Produce Energy?<br>• How Does the Body "Spend" Energy?<br>• Metabolic Changes in ME/CFS<br>• How to Manage and Conserve Energy? (introductory overview; rest and sleep as foundational elements)<br>• Energy: What is my individual energy baseline? (concept of a safe and sustainable level of daily activity)

4	Energy Management – Part I: What is it and why is it important?	• Understand the fundamental principles of energy management.<br>• Identify energy management techniques and tools applicable to daily activities.<br>• Recognise heart rate variability as an indicator of exertion and fatigue.<br>• Identify inappropriate patterns and common mistakes in energy management.	• Energy Management: what is it?<br>• Why is it important to manage energy?<br>• Key Principles of Energy Management<br>• Tools and Techniques for Energy Management<br>• Common mistakes in Energy Management<br>• "Diary of activities and associated symptoms" –home-based self-monitoring task to support the application of energy management principles.
5	Energy Management – Part II: practical techniques for everyday use.	• Use different types of rest and breathing techniques to support recovery and fatigue management.<br>• Use posture and environmental strategies to improve energy efficiency.<br>• Use support devices and practical adaptations in specific situations.<br>• Adapt energy management strategies to PEM days and situations of increased functional limitation.<br>• Apply energy management principles to everyday activities.	• Energy Management: types of rest<br>• Breathing Techniques - optimising functional capacity<br>o Diaphragmatic breathing<br>o Breathing with lips slightly parted<br>o Breathing during exertion<br>• Posture and Positioning in Energy Management<br>• Energy Management Strategies - practical application<br>• Energy Management Strategies - adapting the environment<br>• Energy Management Strategies - support devices<br>• Energy Management Strategies on PEM days<br>• Energy Management Strategies - patients confined to home
6	Nutrition, Hydration, Sleep, and Emotion Management: Other Key Factors for Well-being and Recovery.	• Understand the role of nutrition in supporting wellbeing and recovery in ME/CFS.<br>• Recognise the importance of adequate hydration in people with ME/CFS.<br>• Identify strategies to support sleep quality in people with ME/CFS.<br>• Recognise the impact of emotional regulation on the autonomic nervous system and symptom management in ME/CFS.	• Nutrition – strategies for wellbeing and recovery<br>• Hydration: its role in ME/CFS<br>• Sleep: how to improve sleep quality in people with ME/CFS<br>• Emotion management: implications for the autonomic nervous system and people with ME/CFS
7	Effective Communication: How to Explain Your Needs to Your Family and Healthcare Professionals	• Recognise common communication challenges and needs related to ME/CFS.<br>• Identify strategies to support communication of needs and limitations with family and healthcare professionals.<br>• Recognise the role of effective communication in building supportive relationships and reducing energy burden.	• Communication - Challenges!<br>• Communication with family – Challenges and Strategies<br>• Communication with healthcare professionals – Challenges and Strategies<br>• Effective communication and energy management<br>• Communication - building supportive relationships

8	Long-Term Planning: Building a Sustainable Future with ME/CFS	• Recognise the importance of developing a sustainable and individualised life plan integrating energy management and quality of life strategies.<br>• Identify principles for planning a gradual and safe return to activities such as school or work, when appropriate.<br>• Adapt energy management strategies to situations of increased disease severity or immobility, maintaining a focus on functionality and wellbeing.	• ME/CFS: One Condition, One Change, and One New Perspective<br>o ME/CFS: Understanding the Health-Illness Transition<br>• Building a Sustainable Life Plan<br>o Planning for Return to School<br>o Planning for Return to Work<br>o Activity: Increasing Activity Safely and Sustainably<br>o Severe ME/CFS and Immobility<br>• Energy Management: A Path Under Construction<br>• Sharing participants' experiences (if desired by participants).

Key references informing the development of the intervention

Guidelines, consensus statements and institutional reports

National Institute for Health and Care Excellence. (2021). *Myalgic encephalomyelitis (or encephalopathy)/chronic fatigue syndrome: Diagnosis and management (NICE guideline NG206).* https://www.nice.org.uk/guidance/ng206

Nacul, L., Authier, F. J., Scheibenbogen, C., Lorusso, L., Helland, I. B., Martin, J. A., … Lacerda, E. M. (2021). European Network on Myalgic Encephalomyelitis/Chronic Fatigue Syndrome (EUROMENE): Expert consensus on the diagnosis, service provision, and care of people with ME/CFS in Europe. *Medicina, 57*(5), 510. https://doi.org/10.3390/medicina57050510

Institute of Medicine. (2015). *Beyond myalgic encephalomyelitis/chronic fatigue syndrome: Redefining an illness.* National Academies Press. https://doi.org/10.17226/19012

Carruthers, B. M., Van de Sande, M. I., De Meirleir, K. L., Klimas, N. G., Broderick, G., Mitchell, T., … Stevens, S. (2011). Myalgic encephalomyelitis: International Consensus Criteria. *Journal of Internal Medicine, 270*(4), 327–338. https://doi.org/10.1111/j.1365-2796.2011.02428.x

Wilshire, C. E., Kindlon, T., Courtney, R., Matthees, A., Tuller, D., Geraghty, K., & Levin, B. (2018). Rethinking the treatment of chronic fatigue syndrome—a reanalysis and evaluation of findings from a recent major trial of graded exercise and CBT. *BMC Psychology*, *6*(1), 6. https://doi.org/10.1186/s40359-018-0218-3

World Health Organization. (n.d.). *ICD-11 for mortality and morbidity statistics: Postviral fatigue syndrome.* https://icd.who.int/browse/2024-01/mms/en#569175314

Peer-reviewed scientific evidence informing energy management and rehabilitation in ME/CFS

Sanal-Hayes, N. E. M., McLaughlin, M., Mair, J. L., Ormerod, J., Carless, D., Meach, R., Hilliard, N., Ingram, J., Sculthorpe, N. F., & Hayes, L. D. (2024). *'Pacing' for management of myalgic encephalomyelitis/chronic fatigue syndrome (ME/CFS): A systematic review and meta-analysis*. *Fatigue: Biomedicine, Health & Behavior*. https://doi.org/10.1080/21641846.2024.2433390

Casson, S., Jones, M. D., Cassar, J., Kwai, N., Lloyd, A. R., Barry, B. K., & Sandler, C. X. (2023). *The effectiveness of activity pacing interventions for people with chronic fatigue syndrome: A systematic review and meta-analysis*. *Disability and Rehabilitation, 45*(23), 3788–3802. https://doi.org/10.1080/09638288.2022.2135776

Goudsmit, E. M., Nijs, J., Jason, L. A., & Wallman, K. E. (2012). Pacing as a strategy to improve energy management in ME/CFS: A consensus document. *Disability and Rehabilitation, 34*(13), 1140–1147. https://doi.org/10.3109/09638288.2011.635746

Davenport, T. E., Chu, L., Stevens, S. R., Stevens, J., Snell, C. R., & Van Ness, J. M. (2023). Two symptoms can accurately identify post-exertional malaise in ME/CFS. *Work, 74*(4), 1199–1213. https://doi.org/10.3233/WOR-220554

Ghali, A., Lacout, C., Ghali, M., Gury, A., Delattre, E., Lavigne, C., & Urbanski, G. (2021). Warning signals of post-exertional malaise in ME/CFS: A retrospective analysis of 197 patients. *Journal of Clinical Medicine, 10*(11), 2517. https://doi.org/10.3390/jcm10112517

Koenig, J., & Thayer, J. F. (2019). Heart rate variability in chronic fatigue syndrome and fibromyalgia: A systematic review and meta-analysis. *Current Rheumatology Reports, 21*(9), 44. https://doi.org/10.1007/s11926-019-0831-0

Mohamed, A. Z., Andersen, T., Radovic, S., Del Fante, P., Kwiatek, R., Calhoun, V., … Shan, Z. (2023). Objective sleep measures in chronic fatigue syndrome: A systematic review and meta-analysis. *Sleep Medicine Reviews, 69*, 101771. https://doi.org/10.1016/j.smrv.2023.101771

Educational and clinical web-based resources

Bateman Horne Center. (2022). *ME/CFS crash survival guide* (1st ed.). https://batemanhornecenter.org

Bateman Horne Center. (n.d.). *ME/CFS education.* https://batemanhornecenter.org/education/me-cfs/

Action for ME. (2022). *Pacing for people with M.E.* https://www.actionforme.org.uk/uploads/pdfs/Pacing-for-people-with-ME-2022.pdf

Action for ME. (n.d.). *Sleep and rest.* https://www.actionforme.org.uk/get-information/managing-your-symptoms/sleep-and-rest/

Centers for Disease Control and Prevention. (n.d.). *After the diagnosis: Myalgic encephalomyelitis/chronic fatigue syndrome (ME/CFS).* https://www.cdc.gov/me-cfs/pdfs/after-diagnosis_508.pdf

Emerge Australia. (2023). *Pacing with a heart rate monitor.* https://www.emerge.org.au/wp-content/uploads/2023/11/Pacing-with-a-heart-rate-monitor.pdf

Workwell Foundation. (2023). *Heart rate monitor factsheet.* https://workwellfoundation.org/wp-content/uploads/2023/01/HRM-Factsheet.pdf
