# Supplementary material for: A Short-Term Pacing Intervention in People with Myalgic Encephalomyelitis/Chronic Fatigue Syndrome: A Pilot Study in Portugal
Source: Medicina (Kaunas). 2026 Feb 6;62(2):331. doi: 10.3390/medicina62020331 (PMC12941993; doi:10.3390/medicina62020331)
Supplement: Supplementary file 1 [file medicina-62-00331-s001.zip › medicina-4074475-supplementary/Participant Selection Questionnaire ENGLISH VERSION.pdf]

Presentation of the researcher and the study:

Greetings.

My name is Vânia Ribeiro. I am a Specialist Nurse in Rehabilitation Nursing, and as part of my master's thesis, I am conducting a study aimed at evaluating the effects of a rehabilitation programme focused on energy management in people with ME/CFS. The study will include a home visit, or, if this is not possible, an online visit. All intervention sessions will be conducted online, once a week, between 15 September and 15 November.

I will now ask you some questions to verify whether you meet the inclusion criteria for the study and do not meet any of the exclusion criteria. The interview will take approximately 20–30 minutes. Some of the questions will concern sociodemographic data, while others will address your condition and symptoms.

All information provided will be treated as strictly confidential.

1. Are there any obstacles to participating in online sessions?

   YES ☐ NO ☐

2. Do you have a computer or another electronic device that allows you to participate in online sessions?

   YES ☐ NO ☐

3. Are you willing to continue with the interview and answer questions to determine your eligibility for the study?

   YES ☐ NO ☐

Sociodemographic data and inclusion criteria:

1. Name: ___________________
2. Age: ____________

   (Must be over 18 years of age- YES ☐ NO ☐)

3. Place of residence:
4. How long have you had a diagnosis of ME/CFS?_____________

   (must be longer than 3 months - YES ☐ NO ☐)

5. Do you experience fatigue or tiredness that does not improve with rest?

   YES ☐ NO ☐

6. Do you feel unwell after exertion, with prolonged recovery periods?

   YES ☐ NO ☐

7. Do you experience non-restorative sleep or sleep disturbances?

   YES ☐ NO ☐

8. Do you experience cognitive difficulties (e.g. "brain fog")?

   YES ☐ NO ☐

9. Is your ability to participate in occupational, educational, social, or personal activities significantly reduced compared with your pre-illness level?

   YES ☐ NO ☐

10. Do you have any other diagnosed medical conditions that could explain your symptoms? Have other possible causes been ruled out?

    YES ☐ NO ☐

    a. According to NICE guideline recommendations, the person must meet all of the following criteria for at least 3 months:
       i. Debilitating fatigue that worsens with activity, is not the result of excessive cognitive, physical, emotional, or social exertion, and does not improve substantially with rest.
       ii. PEM - Post-exertional malaise after activity in which the worsening of symptoms: — often has a delay of hours or days at the onset — is disproportionate to the activity — has a prolonged recovery period that can last for hours, days, weeks or longer.
       iii. Non-restorative sleep or sleep disturbances, which may include feeling exhausted, flu-like symptoms or stiffness upon waking, disrupted or shallow sleep, altered sleep patterns, or hypersomnia.
       iv. **Cognitive difficulties (often referred to as "brain fog")**, such as problems with memory, concentration, word-finding, speech, slowed information processing, or multitasking.

b. A significant reduction in the ability to participate in occupational, educational, social, or personal activities compared with pre-illness levels, and,
c. No alternative diagnosis that better explains the symptoms.

11. Application of the Chalder Fatigue Questionnaire - I would like to know whether you have experienced any problems with fatigue/weakness or lack of energy IN THE LAST MONTH. Please answer the following questions using the response that best applies to you. If you have been feeling fatigued for a long time, please compare your current state with the last time you felt well. (Likert 0-3, total score of 0-33).
